# Autoimmune diseases and pregnancy: analysis of a series of cases

**DOI:** 10.1186/s13104-015-1177-x

**Published:** 2015-06-04

**Authors:** Vânia Gomes, Alexandra Mesquita, Carlos Capela

**Affiliations:** Autoimmune Disease Unit, Department of Internal Medicine, Hospital of Braga, Braga, Portugal; Life and Health Sciences Research Institute, School of Health Sciences, University of Minho, Campus Gualtar, 4710-057 Braga, Portugal

**Keywords:** Autoimmune disease, Fertility, Miscarriage, Systemic lupus erythematosus, Antiphospholipid syndrome, Polymyositis, Autoimmune thyroiditis, Behcet’s disease

## Abstract

**Background:**

An autoimmune disease is characterized by tissue damage, caused by self-reactivity of different effector mechanisms of the immune system, namely antibodies and T cells. All autoimmune diseases, to some extent, have implications for fertility and obstetrics. Currently, due to available treatments and specialised care for pregnant women with autoimmune disease, the prognosis for both mother and child has improved significantly. However these pregnancies are always high risk. The purpose of this study is to analyse the fertility/pregnancy process of women with systemic and organ-specific autoimmune diseases and assess pathological and treatment implications.

**Methods:**

The authors performed an analysis of the clinical records and relevant obstetric history of five patients representing five distinct autoimmune pathological scenarios, selected from Autoimmune Disease Consultation at the Hospital of Braga, and reviewed the literature.

**Results:**

The five clinical cases are the following: Case 1–28 years old with systemic lupus erythematosus, and clinical remission of the disease, under medication with hydroxychloroquine, prednisolone and acetylsalicylic acid, with incomplete miscarriage at 7 weeks of gestation without signs of thrombosis. Case 2–44 years old with history of two late miscarriages, a single preterm delivery (33 weeks) and multiple thrombotic events over the years, was diagnosed with antiphospholipid syndrome after acute myocardial infarction. Case 3–31 years old with polymyositis, treated with azathioprine for 3 years with complete remission of the disease, took the informed decision to get pregnant after medical consultation and full weaning from azathioprine, and gave birth to a healthy term new-born. Case 4–38 years old pregnant woman developed Behcet’s syndrome during the final 15 weeks of gestation and with disease exacerbation after delivery. Case 5–36 years old with autoimmune thyroiditis diagnosed during her first pregnancy, with difficult control over the thyroid function over the years and first trimester miscarriage, suffered a second miscarriage despite clinical stability and antibody regression.

**Conclusions:**

As described in literature, the authors found a strong association between autoimmune disease and obstetric complications, especially with systemic lupus erythematosus, antiphospholipid syndrome and autoimmune thyroiditis.

## Background

An autoimmune disease (AID) is characterised by tissue damage, caused by self-reactivity of different effectors mechanisms of the immune system, namely antibodies and T cells. Its occurrence may be associated with genetic and/or environmental predisposition. There is an activation of the adaptive immune response with tissue damage and inflammation in the absence of any infection, exposure to toxins or tumour growth [1]. Although, individually, each AID affects a small number of individuals, as a whole, it is estimated that its prevalence is between 7.6 and 9.4% [2]. All AIDs, to some extent, have implications for fertility and obstetrics. In the general population, about 80% of miscarriages occur in the first 12 weeks of pregnancy and the risk of miscarriage in those under the age of 35 is about 10% while it is about 45% in those over the age of 40 [3].

## Pregnancy and AID

Most AIDs occur frequently in women and should they appear at childbearing age, they pose a potential risk for almost all aspects of reproduction, from fertility to pregnancy itself (Table 1) [4–8]. In the past, it was suggested that women with certain AIDs [particularly systemic lupus erythematosus (SLE)/antiphospholipid syndrome (APS)] should avoid pregnancy. Currently, due to available treatments and specialised care for pregnant women with AID, the prognosis for both mother and child has improved significantly [4, 7, 9]. However, these pregnancies are always high risk, often associated with foetal loss in the first trimester, preeclampsia/eclampsia, intrauterine growth restriction, premature rupture of membranes, placental insufficiency, pre-term birth, caesarean delivery and low birth weight [4, 5, 9]. The overall principle, common to all AIDs, is planning the pregnancy for the remission phase of the disease, in addition to all the care necessary for a successful pregnancy [9].

**Table 1:** Available information on fertility and miscarriage rates in autoimmune diseases (adapted from Carp et al. [5])

Disease	Fertility	Miscarriage
Antiphospolipidic syndrome	New tudies suggest a role played by aAP in infertility pathogenesis	Reported recurrent foetal loss with a 10–19% frequency
Autoimmune thyroiditis	Antibodies associated with increased risk of infertility	Antibodies associated with increased risk of miscarriages (recurrent)
Systemic lupus erythematosus	No increased risk of infertility	aAP are the main miscarriage risk factor and are present in 34%

*aAP* antiphospholipid antibodies.

## Goal

The purpose of this study is to analyse the fertility/pregnancy process of women with AID and assess the pathological and treatment implications.

### Systemic AIDs

#### Systemic lupus erythematosus

SLE is a complex AID with varied clinical manifestations and developments. It is characterised by the presence of antinuclear autoantibodies (ANA), anti-DNA, anti-RNA, anti-Ro/SSA and anti-La/SSB autoantibodies (among others), immune complex deposition and damages to target organs, especially kidneys, skin and joints. It is associated with a significant mortality rate [10]. Immunological mechanisms involved include defects in the removal of immune complexes, apoptosis and antigen presentation. The treatment may be topical (sunscreen and corticosteroids) or systemic, with anti-inflammatory drugs (non-steroidal anti-inflammatory drugs, salicylates) or immunosuppressors (hydroxychloroquine, methotrexate, corticosteroids, cyclophosphamide, mycophenolate mofetil, azathioprine, biological therapy) [11].

#### Antiphospholipid syndrome

APS is defined by the presence of at least one clinical manifestation (venous/arterial thrombosis or obstetric complications) and antiphospholipid antibodies (aAP). aAPs are part of a set of antibodies that recognise negatively charged plasma proteins and include anti-cardiolipin, anti-β-2 glycoprotein and lupus anticoagulant antibodies, among others [4]. It also causes skin and cardiac valves lesions and changes in neurological, renal and haematological functions [12]. This syndrome can be primary or occur in association with other systemic diseases, especially SLE [9]. Many individuals are aAP positive without presenting any symptoms and may develop this syndrome. The prevention of the morbidity associated with APS requires an assessment of the risk of thrombosis and the evaluation of the benefits of antithrombotic therapy, which must be performed individually, taking into account the immunological profile and background [12].

#### Polymyositis (PM)/dermatomyositis (DM)

Polymyositis with/without dermatomyositis is an inflammatory myopathy which begins by symmetrically affecting the proximal muscles, is characterised by an increase in the levels of muscle enzymes (creatine kinase/aldolase), electrophysiological changes and characteristic histological findings. These myopathies may involve the muscles that control breathing and swallowing, the heart (pericarditis, cardiomyopathy and heart failure) or the lungs (complications arising from aspiration, interstitial lung disease and pulmonary hypertension). DM also includes skin changes. While PM is mediated by T cells (CD8+), DM is a vascular disorder, mediated by autoantibodies. The first-line treatment of PM/DM is corticosteroid therapy, and it may call for the administration of other immunosuppressive drugs (azathioprine or methotrexate), to which DM responds better [1].

#### Vasculitis

Vasculitis, an immune-mediated disease, is potentially fatal, especially when it affects medium or large calibre vessels. On the one hand, it can cause aneurysms, ruptures and haemorrhages and on the other, it may lead to luminal stenosis with obstruction, tissue ischemia or infarction. There are three major categories of systemic vasculitis: large-, medium- and small-vessel vasculitis. Its accurate diagnosis is difficult and requires clinical, pathological and laboratory data, crucial for an appropriate diagnosis and therapy [11].

*Behcet’s disease* (BD) is a multisystem vasculitis, characterised by orogenital ulcers, uveitis and skin lesions. It may also affect the gastrointestinal tract, joints, the central nervous system or the cardiovascular system. Venous or arterial thrombosis may occur due to endothelial dysfunction and hypercoagulability. Its diagnosis is primarily clinical, although a positive Pathergy test is a classic indicator of the disease. Its treatment differs and may be topical (corticosteroids) or systemic (corticosteroids, anti-TNF-α) [13–15].

### Organ-specific AIDs

#### Autoimmune thyroiditis (AIT)

90% of non-iatrogenic hypothyroidism in countries without iodine deficiency occurs due to autoimmunity and it is a prevalent condition in women of childbearing age [1, 5, 16]. There are several types of AITs, of which the most noteworthy is Hashimoto’s thyroiditis, characterised by the presence of high levels of antithyroglobulin and anti-thyroid peroxidase antibodies in the presence of hypothyroidism. Available treatment consists of hormone replacement with exogenous thyroxin.

## Methods

The authors performed a systematic literature review and an analysis of the clinical records and relevant obstetric history of 5 patients with AID, representing five distinct AID pathological scenarios, selected from AID Consultation at the Hospital of Braga.

## Results and discussion

The following are the descriptions and analysis of a series of five clinical cases.

### Case 1: systemic lupus erythematosus

A 28 years old Brazilian citizen was diagnosed with SLE in 2010 after the appearance of osteoarticular, vascular, ocular and haematological signs/symptoms. aAP were all negative and the liver was never affected. After stabilisation, she was subjected to a regular treatment with prednisolone (10 mg/day) and hydroxychloroquine (400 mg/day)—with periods of intensification of the immunosuppressive dosage due to discoid lupus lesions. The last exacerbation of skin lesions was on December 2012. She first planned pregnancy in March 2013, under medication with hydroxychloroquine 400 mg/day, prednisolone 2.5 mg/day and acetylsalicylic acid 100 mg/day but suffered incomplete miscarriage at 7 weeks gestation, requiring uterine curettage. A histopathological analysis of the abortion material revealed no signs of thrombosis. There was no clinical or laboratory exacerbation of SLE during or after pregnancy, apart from the skin lesions previously described.

#### Discussion

SLE associated with pregnancy can lead to maternal, obstetric and foetal problems (related to the AID). It is agreed that the maternal and foetal prognosis is better when the disease is in remission/quiescent for at least 6 months prior to conception (Table 2) [17]. This patient was in clinical remission at least since 2011. The presence of aAP, anti-SSA/Ro or anti-SSB/La antibodies, hypertension or renal impairment, situations that comprise a greater risk of complications, were excluded. During the 7 weeks of gestation, there was no disease activity or lupus flares, although inavailable literature, a greater incidence of acute exacerbations during pregnancy is described [18], nor were there any complications commonly present in pregnant women with SLE (hypertension, preeclampsia/eclampsia, premature rupture of membranes and gestational diabetes, which usually appear at later stages [19]). The patient eventually had a miscarriage in the first trimester, a common complication in SLE [4, 5, 9]. Histopathology revealed no signs of thrombosis, which safely excludes vascular phenomena associated with APS and/or vasculitis, which could foretell a bleaker prognosis for a new pregnancy. In terms of treatment (Table 3), corticosteroids, and hydroxychloroquine were properly maintained. The abrupt discontinuation of hydroxychloroquine is one of the main causes of exacerbation of the disease during pregnancy [20].

**Table 2:** Contraindications for pregnancy in patients with systemic lupus erythematosus (adapted from Andreoli et al. [9])

1. Severe pulmonary hypertension (systolic BP >50 mmHg or symptomatic)
2. Heart failure
3. Severe restrictive lung disease
4. Mild/severe chronic liver failure
5. Treatment with high dosages of corticosteroids
6. Exacerbation in the last 6 months
7. Previous severe preeclampsia or HELLP syndrome, despite treatment with aspirin and heparin

*BP* blood pressure, *HELLP* hemolysis elevated liver enzymes low platelet count.

**Table 3:** Drugs used in the treatment of autoimmune diseases/compatibility with pregnancy (adapted from Andreoli et al. [9])

Drug	FDA category	Permitted during pregnancy	Notes
Prednisolone	B	Allowed	Associated to medical/obstetric complications (maternal diabetes, preeclampsia, premature rupture of membranes)
NSAID	B/D	Allowed; avoid from 3rd trimester onward	Risk of premature closure of the arterial duct in the 3rd trimester
Hydroxychloroquine	C	Allowed	Discontinuation during pregnancy is associated with SLE exacerbations
Azathioprine	D	Allowed	In the smallest therapeutic dosage, if benefits outweigh risks
Cyclosporine	C	Allowed	
Tacrolimus	C	Allowed	
Sulfasalazine	B	Allowed	
Methotrexate	X	Discontinue 3–6 months before a planned pregnancy	
Cyclophosphamide	D	Discontinue at least 3 months before a planned pregnancy	
Mycophenolatemofetil	D	Discontinue at least 6 weeks before a planned pregnancy	
Warfarin	D	Discontinue after positive pregnancy test	Can be used while breastfeeding
LMWH	B	Allowed	Used as primary prevention of thrombotic events during the puerperal period
IVIG	C	Allowed	
Rituximab	C	Discontinue at least 6–12 months before a planned pregnancy	
Belimumab	C	Discontinue at least 4 months before a planned pregnancy	

FDA-assigned pregnancy categories (The United States Food and Drug Administration): *A* controlled studies in humans have failed to demonstrate a risk to the foetus, *B* no evidence of risk for the human species, *C* Teratogenic—risk to humans cannot be excluded, *D* clear evidence of risk to the human foetus—risk is acceptable in a situation of very high risk for the pregnant woman, in lack of safer alternatives, *X* Drugs considered unsafe during pregnancy.

*NSAID* nonsteroidal anti-inflammatory drugs, *LMWH* low-molecular-weight heparin, *IVIG* intravenous immunoglobulin, *SLE* systemic lupus erythematosus.

### Case 2: antiphospolipid syndrome

A 44 years old, Portuguese citizen, had a first miscarriage at 6 months gestation, in 1992, and a second miscarriage at 8 months gestation, in 1993. Her first and single delivery occurred in 1994 and was a preterm of 33 weeks who required hospitalisation in Neonatology. She had also a history of deep venous thrombosis, with a first episode in 2004 and others in 2006 (including brachial region), having subsequently initiated treatment with oral anticoagulants, which was suspended in late 2012 due to methorragias. In January 2013, she was admitted to the hospital due to acute myocardial infarction with extensive thrombus in the middle right coronary artery. Tests were positive for anti-β-2-glycoprotein (IgG/IgM), anti-cardiolipin antibodies (IgG/IgM) and ANAs, which led to an APS diagnosis and resumption of oral anticoagulant medication.

#### Discussion

APS is probably the AID with the most implications for pregnancy. The reason for the referral and follow-up of this patient to an AID consultation was not related to the pregnancy, but her obstetric history helps support the diagnosis. Generally, after the diagnosis of APS and during pregnancy, anti-thrombotic therapy is based on low-molecular-weight heparin (Table 3) [9]. In this patient’s case, it should be pointed out that, after the second miscarriage, APS could have been diagnosed at the first thrombotic event.

### Case 3: polymyositis

A 31 years old Portuguese citizen with a history of PM, diagnosed in 2008 based on a clinical scenario of polyarthralgia and myalgia with myositis and a biopsy which confirmed the diagnosis. She had a normal caesarean delivery in 2006 and a current pregnancy progressing uneventfully. PM was diagnosed after the first pregnancy. She was treated with high doses of prednisolone for 1 year, and was then put on azathioprine. After 3 years of complete remission and due to her (informed) decision to get pregnant, she began a full weaning from azathioprine, having stabilised with 10 mg/day prednisolone. An angiography was performed and excluded pulmonary hypertension. She then became pregnant and delivered a full term healthy new-born in August 2013.

#### Discussion

Given the low incidence (1–9 cases per million) of PM/DM, its implications for pregnancy are poorly known and existing literature consists of mere descriptions of cases or series of cases [21]. Fertility is still subject to speculation. The trend towards lower fertility may be more related to prior treatment history [22]. The severity of the autoimmune inflammatory activity affects the foetal prognosis. Complete remission of the disease translates into a better outcome of pregnancy [23]; otherwise, abortion rates are almost always above 50% [24]. This patient’s stability and remission were evident, which contributed to the good obstetric development described. Pulmonary hypertension may be one of the manifestations (although rare, ~5%) of PM/DM [25], constituting, besides an activity of the disease, a risk factor for pregnant women (10–40% mortality). A decision was made to study the presence of this manifestation, despite the absence of any cardio- or respiratory symptoms. After it was excluded, pregnancy was safely advised. Unlike SLE, pregnancy does not induce exacerbations of PM. In terms of treatment (Table 3), although azathioprine is allowed during pregnancy, it is the team who decides on whether to maintain or exclude it [26]; in this case, the decision was to replace it with an appropriate dosage of corticosteroids.

### Case 4: vasculitis—Behcet’s disease

Thirty-eight years old Portuguese citizen with an obstetric history of 3G 2P 1A. She had a first delivery in 2004 that occurred uneventful. In 2011, she had a miscarriage at 7 weeks gestation, with no pathological analysis of the abortion material. The third pregnancy was unplanned. During the final 15 weeks of gestation, BD was diagnosed due to erythema nodosum on limbs and genital ulcers (minor criteria) and recurrent oral thrush (major criteria), with a 2-year evolution, exacerbated during pregnancy. Throughout the entire pregnancy, she was only treated with topical betamethasone to control oral thrush. The delivery was eutocic, preterm (36 weeks), due to premature rupture of membranes. The new-born infant required hospitalisation in Neonatology and was discharged on day 7. Due to persistent oral thrush and postpartum genital ulcers, systemic corticosteroid therapy was initiated (5 mg/day).

#### Discussion

The BD diagnosis is sometimes suggested by the gynaecologist due to the presence of genital ulcers, being oral thrush usually underestimated [27, 28]. It is a widespread opinion that pregnancy does not affect the natural development of BD and vice versa [29], except in rarer conditions associated with a worse prognosis for pregnancy (vasculopathy, thrombosis and pulmonary hypertension), where it has been shown to affect the placenta at an early stage [15]. We did not find in the literature a description of the relationship between BD and preterm delivery as in this case, but we found other foetal problems [30]. Immunosuppressive therapy, as in the other cases studied, may also have implications. BD, and especially severe oral thrush, have an undetermined behaviour during pregnancy, so the treatment must be adjusted to each individual using the risk/benefit principle. This patient’s symptoms were only oral thrush, and so the treatment adopted was topical. No systemic therapy was administered postpartum, considering the absence of other manifestations (vascular/uveitis).

### Case 5: autoimmune thyroiditis

Thirty-six years old Portuguese citizen, with a history of AIT (Hashimoto’s disease) diagnosed in 2006 during her first pregnancy. She was medicated with levothyroxine in widely variable doses to achieve clinical/laboratory goals. Her obstetric history up to the first consultation was 2G 1P 1A. The first pregnancy had no complications but it was eutocic and preterm (34 weeks) and an miscarriage occurred during the first trimester in 2012. She was then referred to the AID consultation for pathology review and due to very high levels of antithyroglobulin and anti-thyroid peroxidase antibodies and difficult control over the thyroid function. After systemic AID was excluded and 4 months of clinical stability with antibody regression, she became pregnant. A new miscarriage occurred at 7 weeks gestation.

#### Discussion

Upon the diagnosis of organ-specific AID (Hashimoto thyroiditis, type 1 diabetes, vitiligo), associated systemic diseases must be ruled out. Clinical immunology studies have shown that, during pregnancy, initially high levels of antithyroid antibodies fall abruptly due to the immune tolerance environment [31]. However, AIT is associated with higher rates of infertility and early miscarriages, due to the associated hormonal changes and instability and higher age (the average age of the study groups is above normal childbearing age, which is associated with increased obstetric risk), as is the case with this patient. The presence of antithyroid antibodies may react against the structures of the placenta or fertilized egg and cause problems in embryo implantation [32, 33]. This patient’s hormonal instability and age were possible causes for the miscarriages.

## Conclusions

As described in literature, we found a strong association between AIDs and obstetric complications, especially with SLE, APS and AIT—of the 12 pregnancies studied, 50% resulted in miscarriage and 25% in preterm birth. However, it is important to point out that these cases were already particularly complicated, as they concern women who were being followed in specific AID consultations, which excludes simpler cases that do not require such specialised care.

## Abbreviations

AID: Autoimmune disease
SLE: Systemic lupus erythematosus
APS: Antiphospholipid syndrome
ANA: Antinuclear autoantibodies
aAP: Antiphospholipid antibodies
PM: Polymyositis
DM: Dermatomyositis
BD: Behcet’s disease
AIT: Autoimmune thyroiditis.
